# Draft Genome Sequence of Enterobacter sp. Strain EA-1, an Electrochemically Active Microorganism Isolated from Tropical Sediment

**DOI:** 10.1128/genomeA.00111-18

**Published:** 2018-03-01

**Authors:** Lucinda E. Doyle, Rohan B. H. Williams, Scott A. Rice, Enrico Marsili, Federico M. Lauro

**Affiliations:** a Singapore Centre for Environmental Life Sciences Engineering, Nanyang Technological University, Singapore; b Singapore Centre for Environmental Life Sciences Engineering, National University of Singapore, Singapore; c ithree Institute, University of Technology, Sydney, Australia; d School of Biological Sciences, Nanyang Technological University, Singapore; e School of Chemical and Biomedical Engineering, Nanyang Technological University, Singapore; f Asian School of the Environment, Nanyang Technological University, Singapore

## Abstract

Enterobacter sp. strain EA-1 is an electrochemically active bacterium isolated from tropical sediment in Singapore. Here, the annotated draft genome assembly of the bacterium is reported. Whole-genome comparison indicates that Enterobacter sp. EA-1, along with a previously sequenced Enterobacter isolate from East Asia, forms a distinct clade within the Enterobacter genus.

## GENOME ANNOUNCEMENT

Members of the genus Enterobacter belong to the class *Gammaproteobacteria* and are rod-shaped, Gram-negative, motile, facultative anaerobes. They are commonly found in soil, water, sewage, and the intestines of animals and humans and have been associated with nosocomial infections (1). Enterobacter sp. strain EA-1 was isolated from tropical sediment in Singapore during an electrochemical enrichment and was found to be capable of extracellular electron transfer (2).

Genomic DNA was purified from 1 ml of an overnight culture grown in Luria-Bertani (LB) broth at room temperature and with shaking at 150 rpm. The Wizard genomic DNA purification kit (Promega, USA) was used according to the protocol for Gram-negative bacteria supplied by the manufacturer.

The draft genome sequence was determined by shotgun sequencing on a MinION Mk1B sequencer (Oxford Nanopore, UK) using R9 chemistry and library protocol SQK-LSK108. Raw sequencing data were assembled using Canu version 1.3 (3), resulting in a single contig which was further refined using nanopolish version 0.7.1 (4), manually trimmed for overlaps at the ends, and automatically annotated using the NCBI Prokaryotic Genome Annotation Pipeline (5).

The draft genome of EA-1 is composed of 5,145,523 bp, with an average G+C content of 54.56%, and it contains 81 tRNAs, 22 rRNAs organized in 7 operons, and 1 transfer-messenger RNA (tmRNA). A comparison of the 16S rRNA gene sequence revealed >98% identity to the 16S rRNA genes of Enterobacter cloacae ATCC 13047, Enterobacter cloacae SBP-8, and Enterobacter sp. strain FY-07. However, a whole-genome comparison indicated an average nucleotide identity (6) of 78.12% compared to E. cloacae ATCC 13047 (NCBI accession number CP001918), 78.09% compared to E. cloacae SBP-8 (NCBI accession number CP016906), and 93.38% compared to FY-07 (NCBI accession number CP012487), suggesting that EA-1 forms, together with FY-07, a distinct clade within the Enterobacter genus related to but separate from E. cloacae.

In-detail genome alignments between EA-1 and FY-07 using the Artemis Comparison Tool (7) showed that the genes associated with bacterial cellulose (BC) in FY-07, a highly effective producer of the polysaccharide (8), are also present in strain EA-1, with open reading frame (ORF) nucleotide identities ranging from 82 to 95%. Pairwise global alignment was conducted at the EMBL-EBI website (http://www.ebi.ac.uk/Tools/psa/) using the EMBOSS Needle tool (Needleman-Wunsch algorithm). In FY-07, these genes are arranged in three BC-producing operons: *bcsI*, *bcsII*, and *bcsIII* (9). In *bcsI*, the FY-07 locus tags are as follows, with the corresponding EA-1 locus tags in parentheses: AKI40_0196 (CWS02_01210), AKI40_0197 (CWS02_01215), AKI40_0198 (CWS02_01220), AKI40_0199 (CWS02_01225), AKI40_0200 (CWS02_01230), AKI40_0201 (CWS02_01235), and AKI40_0202 (CWS02_01240). For *bcsII*, the FY-07 and corresponding EA-1 locus tags (in parentheses) are AKI40_0206 (CWS02_01260), AKI40_0207 (CWS02_01265), AKI40_0208 (CWS02_01270, CWS02_01275, and CWS02_01280), AKI40_0209 (CWS02_01285), AKI40_0210 (CWS02_01290), and AKI40_0211 (CWS02_01295). In the case of the EA-1 locus tag corresponding to FY-07’s AKI40_0208, multiple ORFs were stitched together during nucleotide alignment in order to identify the complete homologous gene sequence which was interrupted due to frameshifting errors associated with the sequencing technology used. For *bcsIII*, the FY-07 and corresponding EA-1 locus tags (in parentheses) are AKI40_0894 (CWS02_05060), AKI40_0893 (CWS02_05055), AKI40_0892 (CWS02_05050), and AKI40_0891 (CWS02_05045). For other BC-related genes (not operon-associated), the FY-07 and corresponding EA-1 locus tags are AKI40_0203 (CWS02_01245), AKI40_0204 (CWS02_01250), and AKI40_0205 (CWS02_01255).

### Accession number(s).

The whole-genome shotgun project was deposited in NCBI under the accession number CP025776.
